# Supplementary material for: Preclinical Multi-Omic Assessment of Pioglitazone in Skeletal Muscles of Mice Implanted with Human HER2/neu Overexpressing Breast Cancer Xenografts
Source: Cancers (Basel). 2024 Oct 29;16(21):3640. doi: 10.3390/cancers16213640 (PMC11544806; doi:10.3390/cancers16213640)
Supplement: Supplementary file 1 [file cancers-16-03640-s001.zip › cancers-3205215-supplementary.pdf]

Supplementary Table S1. Sample sizes used for all components of study. For each group, values indicate the number of samples included in analysis. PioTx = pioglitazone-treated.

	Variable	Study Group		
		PioTx	Vehicle	Naive NSG
Metabolomics/Lipidomics	All	6	5	4
Whole-animal indirect calorimetry	All	5	4	
Bulk RNA-seq	All	6	5	
Weights	TA	12	9	
	EDL	12	9	
	Gastrocnemius	12	9	
	Soleus	12	9	
	Body Mass	6	5	
Muscle Contractile Properties	L_o	6	5	
	CSA	6	5	
	Peak Twitch	6	5	
	Peak CT	6	5	
	Peak RFD	6	5	
	Peak ½ RT	6	5	
	Peak RR	6	5	
	Peak Tetanus	6	5	
	Twitch/CSA	6	5	
	Tetanus/CSA	6	5	
	Twitch/Tetanus	6	5	
Force-Frequency	Force (@ each frequency)	6	5	
Fatigue	Peak Force per Rep	6	5	

Supplementary Table S2. *Ex vivo* isometric contractile metrics for EDL. Values reported as mean ± SD. L_o = optimal length, CSA = cross-sectional area, mN = milliNewton.

EDL Isometrics			
	**Vehicle**	**Pioglitazone**	***p*-value**
EDL L_o (mm)	10.3 ± 0.7	10.6 ± 0.5	0.4951
EDL CSA (mm^2)	1.2 ± 0.3	1.4 ± 0.2	0.2583
Twitch (mN)	28.6 ± 7.2	25.5 ± 4.3	0.4013
Twitch (mN * CSA^{-1})	25.0 ± 6.4	18.8 ± 4.3	0.0871
Contraction time (ms)	20.0 ± 7.1	23.3 ± 8.2	0.4927
Rate of force development (mN * s^{-1})	1082 ± 318.3	910.5 ± 116.6	0.2478
½ Relaxation time (ms)	28.0 ± 4.5	31.7 ± 4.1	0.1889
Rate of relaxation (mN * s^{-1})	-702.7 ± 216.9	-555.5 ± 144.1	0.2101
Tetanus (mN)	156.2 ± 19.4	160.0 ± 27.0	0.7968
Tetanus (mN * CSA^{-1})	136.8 ± 24.4	117.3 ± 24.5	0.2192
P_{min} (mN)	27.62 ± 5.249	24.48 ± 7.375	0.7387
P_{max} (mN)	163.0 ± 8.140	165.8 ±10.89	0.8393
K_f (Hz)	43.12 ± 3.852	41.71 ± 4.941	0.8308
Hill Slope	2.535 ± 0.5380	2.525 ± 0.7068	0.9914

Supplementary Table S3. *Ex vivo* isometric contractile metrics for soleus. Values reported as mean ± SD. L_o = optimal length, CSA = cross-sectional area, mN = milliNewton.

Soleus Isometrics			
	**Vehicle**	**Pioglitazone**	***p*-value**
Soleus L_o (mm)	8.5 ± 0.5	8.8 ± 0.7	0.4311
Soleus CSA (mm^2)	1.0 ± 0.3	0.9 ± 0.3	0.7512
Twitch (mN)	7.3 ± 2.3	9.9 ± 3.7	0.2096
Twitch (mN * CSA^{-1})	8.9 ± 6.3	12.4 ± 5.9	0.3700
Contraction time (ms)	52.0 ± 11.0	61.7 ± 11.7	0.1938
Rate of force development (mN * s^{-1})	135.8 ± 49.3	151.8 ± 37.0	0.5546
½ Relaxation time (ms)	74.0 ± 28.8	106.7 ± 19.7	0.0524
Rate of relaxation (mN * s^{-1})	-51.9 ± 53.3	-135.4 ± 210.4	0.4135
Tetanus (mN)	76.6 ± 26.7	90.4 ± 21.4	0.3652
Tetanus (mN * CSA^{-1})	90.0 ± 55.0	109.3 ± 39.3	0.5127
P_{min} (mN)	4.654 ± 13.26	2.233 ± 21.03	0.9228
P_{max} (mN)	79.28 ± 12.04	91.78 ± 7.274	0.4481
K_f (Hz)	28.12 ± 8.686	16.76 ± 5.934	0.2567
Hill Slope	1.876 ± 1.295	1.763 ± 0.9354	0.9404

Supplementary Table S4. Top 5 up- and downregulated pathways in PioTx vs. vehicle for all gene set sources. All adjusted (Adj.) *p*-value significant pathways can be found in Supplementary Data 5-9.

**PioTx vs. Vehicle**					
Source	Direction	Pathway	Statistic	# of genes	Adj. Pval
GO Bio	Down	GO:0030334 regulation of cell migration	-4.6764	491	0.006
		GO:0061061 muscle structure development	-4.5051	420	0.0069
		GO:0060537 muscle tissue development	-4.3546	257	0.01
		GO:0030335 positive regulation of cell migration	-4.177	294	0.014
		GO:0040017 positive regulation of locomotion	-4.121	306	0.014
	Up	GO:0042776 proton motive force-driven mitochondrial ATP synthesis	7.2518	61	< 0.001
		GO:0042773 ATP synthesis coupled electron transport	7.2144	70	< 0.001
		GO:0022904 respiratory electron transport chain	7.0116	86	< 0.001
		GO:0042775 mitochondrial ATP synthesis coupled electron transport	7.0104	68	< 0.001
		GO:0009060 aerobic respiration	6.9121	162	< 0.001
GO Cell	Down	GO:0031252 cell leading edge	-3.8918	252	0.016
		GO:0015629 actin cytoskeleton	-3.6586	289	0.016
		GO:0099512 supramolecular fiber	-3.6304	488	0.016
		GO:0099081 supramolecular polymer	-3.6295	490	0.016
		GO:0042641 actomyosin	-3.393	61	0.041
	Up	GO:0098798 mitochondrial protein-containing complex	10.7536	272	< 0.001
		GO:0005743 mitochondrial inner membrane	9.7133	418	< 0.001
		GO:0098800 inner mitochondrial membrane protein complex	9.4954	137	< 0.001
		GO:0019866 organelle inner membrane	9.0888	446	< 0.001
		GO:0070469 respirasome	8.3884	88	< 0.001
GO Mol	Down	GO:0003779 actin binding	-3.9998	257	0.019
		GO:0008134 transcription factor binding	-3.3466	405	0.049
		GO:0019900 kinase binding	-3.3391	491	0.049
		GO:0061629 RNA polymerase II-specific DNA-binding transcription factor binding	-3.3022	249	0.049
		GO:0019901 protein kinase binding	-3.201	439	0.049
	Up	GO:0016491 oxidoreductase activity	5.1059	358	< 0.001
		GO:0015453 oxidoreduction-driven active transmembrane transporter activity	4.867	28	0.002
		GO:0009055 electron transfer activity	4.5014	49	0.002
Reactome	Up	R-MMU-163200 Respiratory electron transport ATP synthesis by chemiosmotic coupling and heat production by uncoupling proteins	8.2408	113	< 0.001
		R-MMU-1428517 The citric acid TCA cycle and respiratory electron transport	7.9483	154	< 0.001
		R-MMU-611105 Respiratory electron transport	7.4995	94	< 0.001
		R-MMU-6799198 Complex I biogenesis	6.5292	54	< 0.001
		R-MMU-5368287 Mitochondrial translation	5.3255	88	< 0.001
KEGG	Up	Path:mmu00190 Oxidative phosphorylation	7.4728	114	< 0.001
		Path:mmu04714 Thermogenesis	5.294	187	< 0.001
		Path:mmu05415 Diabetic cardiomyopathy	4.7908	160	< 0.001
		Path:mmu05208 Chemical carcinogenesis-reactive oxygen species	4.0763	167	0.002
		Path:mmu04723 Retrograde endocannabinoid signaling	4.0202	76	0.0026

Supplementary Table S5. Abbreviations and corresponding complete names for all identified lipid classes.

**BA**	bile acids	**CAR**	carnitines	**Cer-AP**	ceramide alpha-hydroxy fatty acid-phytosphingosine
**Cer-AS**	ceramide alpha-hydroxy fatty acid-sphingosine	**Cer-NDS**	ceramide non-hydroxy fatty acid-dihydrosphingosine	**Cer-NP**	ceramide non-hydroxy fatty acid-phytosphingosine
**Cer-NS**	ceramide alpha-hydroxy fatty acid-sphingosine	**Cholesterol**	cholesterol	**CoQ**	coenzyme Q
**FFA**	free fatty-acids	**HexCer-AP**	hexosylceramide alpha-hydroxy fatty acid-phytosphingosine	**HexCer-NS**	Hexosylceramide non-hydroxy fatty acid-sphingosine
**LNAPE**	N-acyl-lysophosphatidylethan olamine	**LPA**	lysophosphatidic acid	**LPC**	lysophosphatidylcholine
**LPC-O**	alkyl-lysophosphatidylcholine	**LPE**	lysophosphatidylethanolamine	**LPE-P**	alkenyl-lysophosphatidylethan olamine
**LPG**	lysophosphatidylglycerol	**LPI**	lysophosphatidylinositol	**LPS**	lysophosphatidylserine
**MG**	monoacylglycerol	**PC**	phosphatidylcholine	**PC-O**	alkyl-phosphatidylcholine
**PE**	phosphatidylethanolamine	**PE-O**	alkyl-phosphatidylethanolamine	**PE-P**	alkenyl-phosphatidylethanola mine
**PG**	phosphatidylglycerol	**PI**	phosphatidylinositol	**PMeOH**	phosphatidylmethanol
**PS**	phosphatidylserine	**SM**	sphingomyelin	**SPH**	sphingosine
**TG**	triacylglycerol				

Supplementary Table S6. Weight in milligrams of all isolated muscles from all groups of mice. Muscles isolated were extensor digitorum longus (EDL), soleus, tibialis anterior (TA), and gastrocnemius (Gastroc). Missing TA length values and corresponding normalizations were due to fractured tibias and not included in analysis. TL: tibia length (mm). / TL: muscle weight divided by tibia length. PIO: tumor-bearing and pioglitazone treated. VEH: tumor-bearing and vehicle treated.

	**ID**	**Limb**	**TL (mm)**	**EDL**		**Soleus**		**TA**		**Gastroc**	
				**mg**	**/ TL**	**mg**	**/ TL**	**mg**	**/ TL**	**mg**	**/ TL**
PIO	PIO-1	R	17.84	8	0.448	7	0.392	43	2.410	118	6.614
		L	17.77	10	0.563	8	0.450	44	2.476	139	7.822
	PIO-2	R	18.1	7	0.387	6	0.331	43	2.376	126	6.961
		L	18.4	9	0.489	7	0.380	43	2.337	156	8.478
	PIO-3	R	18.26	7	0.383	6	0.329	49	2.683	143	7.831
		L	18.33	9	0.491	8	0.436	47	2.564	156	8.511
	PIO-4	R	18.45	7	0.379	8	0.434	47	2.547	118	6.396
		L	18.43	8	0.434	10	0.543	45	2.442	88	4.775
	PIO-5	R	18.27	8	0.438	7	0.383	49	2.682	122	6.678
		L	18.32	10	0.546	3	0.164	47	2.566	173	9.443
	PIO-6	R	17.8	5	0.281	4	0.225	32	1.798	126	7.079
		L	18.9	7	0.370	8	0.423	35	1.852	109	5.767
VEH	VEH-1	R	17.54	6	0.342	9	0.513	39	2.223	114	6.499
		L	17.06	6	0.352	11	0.645	38	2.227	119	6.975
	VEH-2	R	17.2	5	0.291	7	0.407	46	2.674	144	8.372
		L	/	5	/	13	/	49	/	146	/
	VEH-3	R	18.34	8	0.436	5	0.273	41	2.236	127	6.925
		L	18.51	8	0.432	9	0.486	39	2.107	130	7.023
	VEH-4	R	18.14	6	0.331	6	0.331	41	2.260	140	7.718
		L	18.18	11	0.605	10	0.550	41	2.255	141	7.756
	VEH-5	R	18.0	4	0.222	4	0.222	33	1.833	107	5.944
		L	17.87	6	0.336	6	0.336	29	1.623	106	5.932

Supplementary Figure S1. STR profile comparison of original tumor with passaged injected HER2/neu overexpressing PDX tumor. (**a**) STR profile of original patient tumor sample. (**b**) STR profile of passaged injected tumor sample.

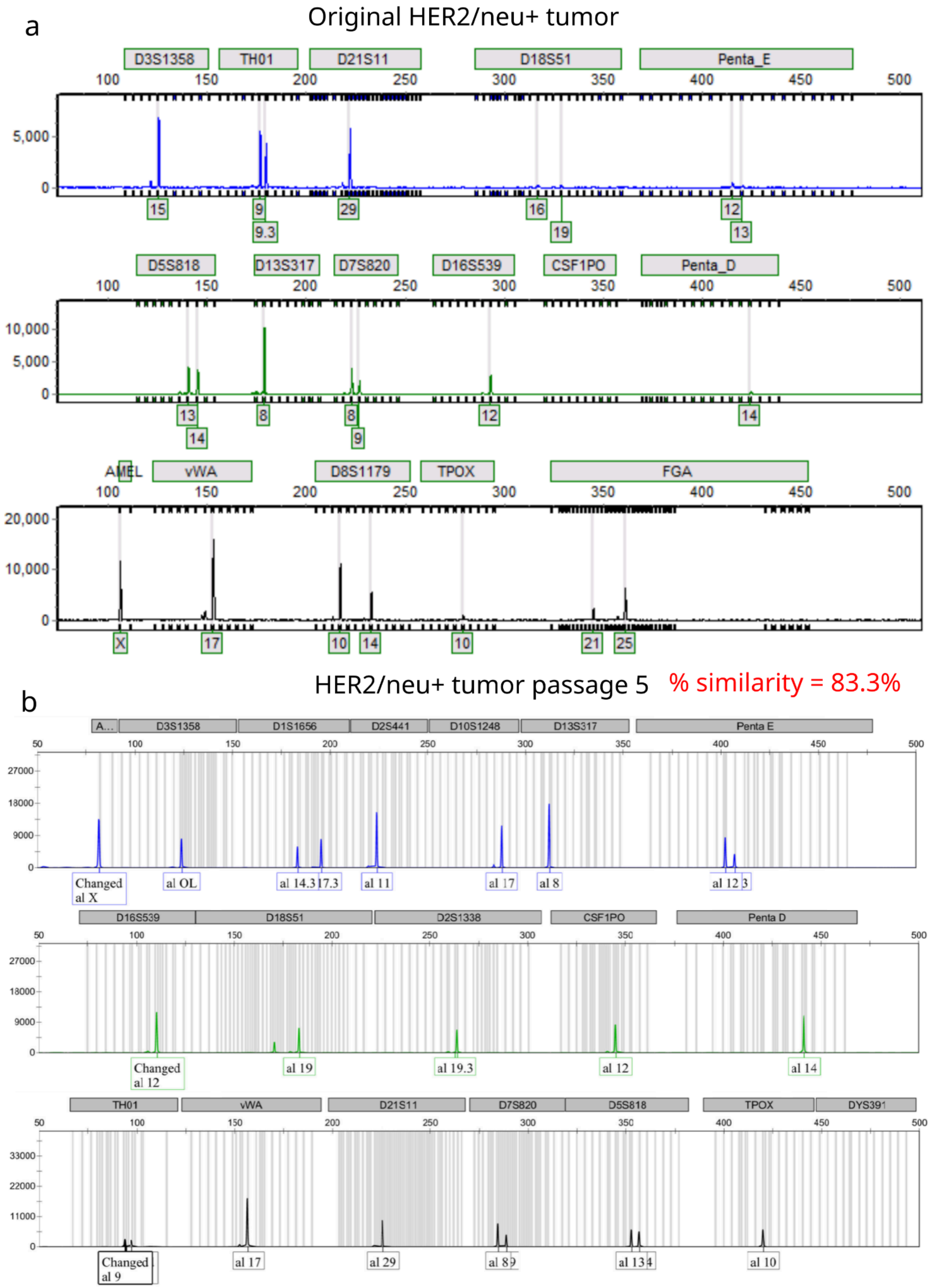

Supplementary Figure S2. Additional bulk RNA-seq analysis. (**a**) Bioanalyzer image of all samples (B1-H1, A2-D2). A1 is the ladder. Note: figure includes samples that are not part of this study (E2-H2, A3-C3). (**b**) Total raw read counts in millions for pioglitazone
(salmon) and vehicle (blue). (**c**) Standard deviation of all genes and top 1000 (red dotted line). (**d**) SCREE plot of principal component expected variation. (**e**) Lollipop plot of k-means cluster 5 showing enrichment of PPAR signaling pathway. (**f**) GSEA enrichment of the

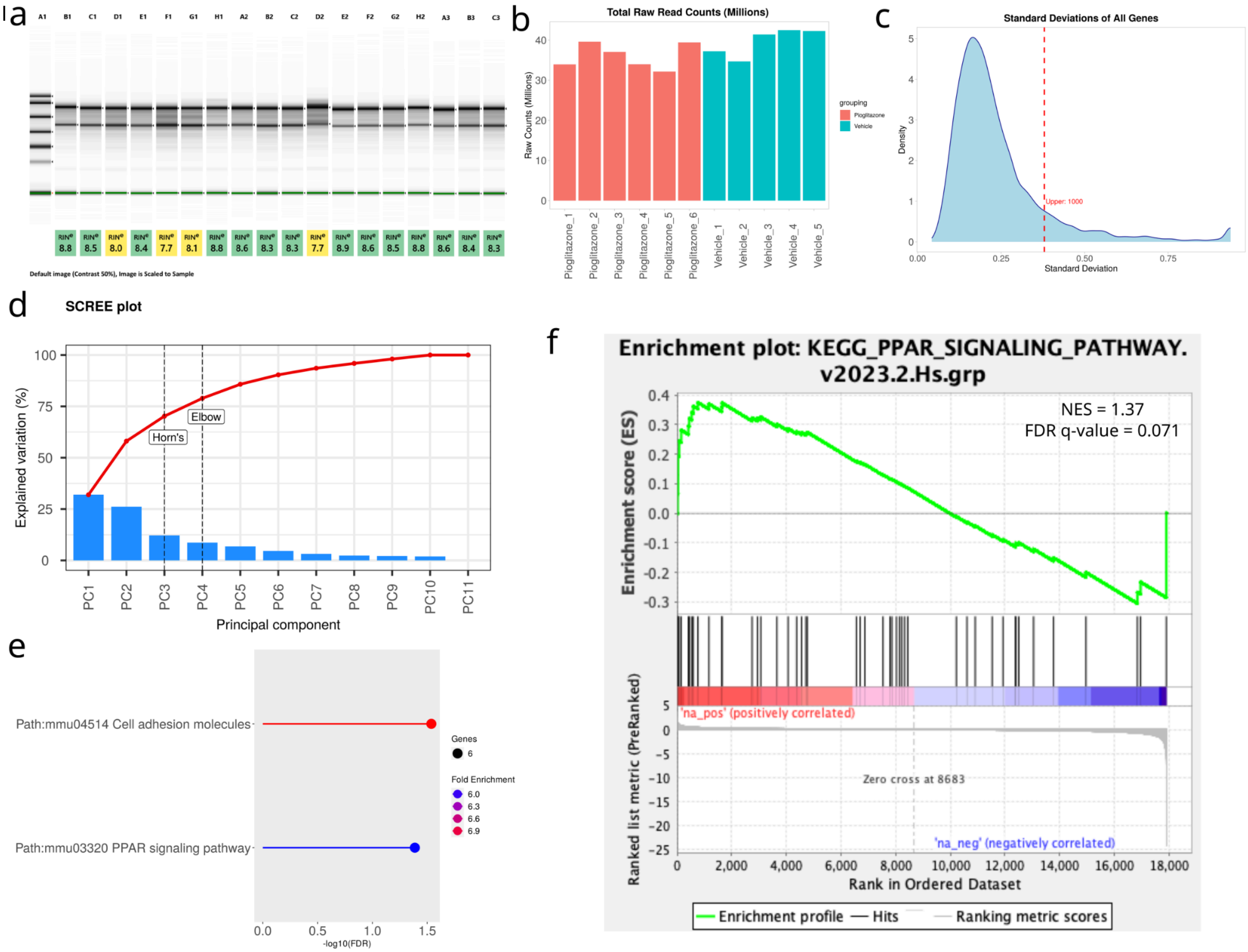

Supplementary Figure S3. Quality control (QC) metrics for quantitative lipidomics. (**a**) Ring plot describing proportion of each compound class combined from all samples. (**b**) Pearson correlation analysis of QC samples. The |r| values in upper right squares represent correlation between QC samples, the closer to 1 the better. (**c**) Two-dimensional principal component analysis (PCA) plot including QC samples. (**d**) Coefficient of variation (CV) plot for QC and sample groups. Horizontal axis represents CV value and vertical axis represents the percent of peaks (proportion of metabolites). (**e**) Principal component 1 (PC1) variation of all samples. Horizontal axis represents injection order of samples and vertical axis represents standard deviation (SD) of PC1 score. (**c,e**) Abbreviation CON refers to Naive NSG mice.

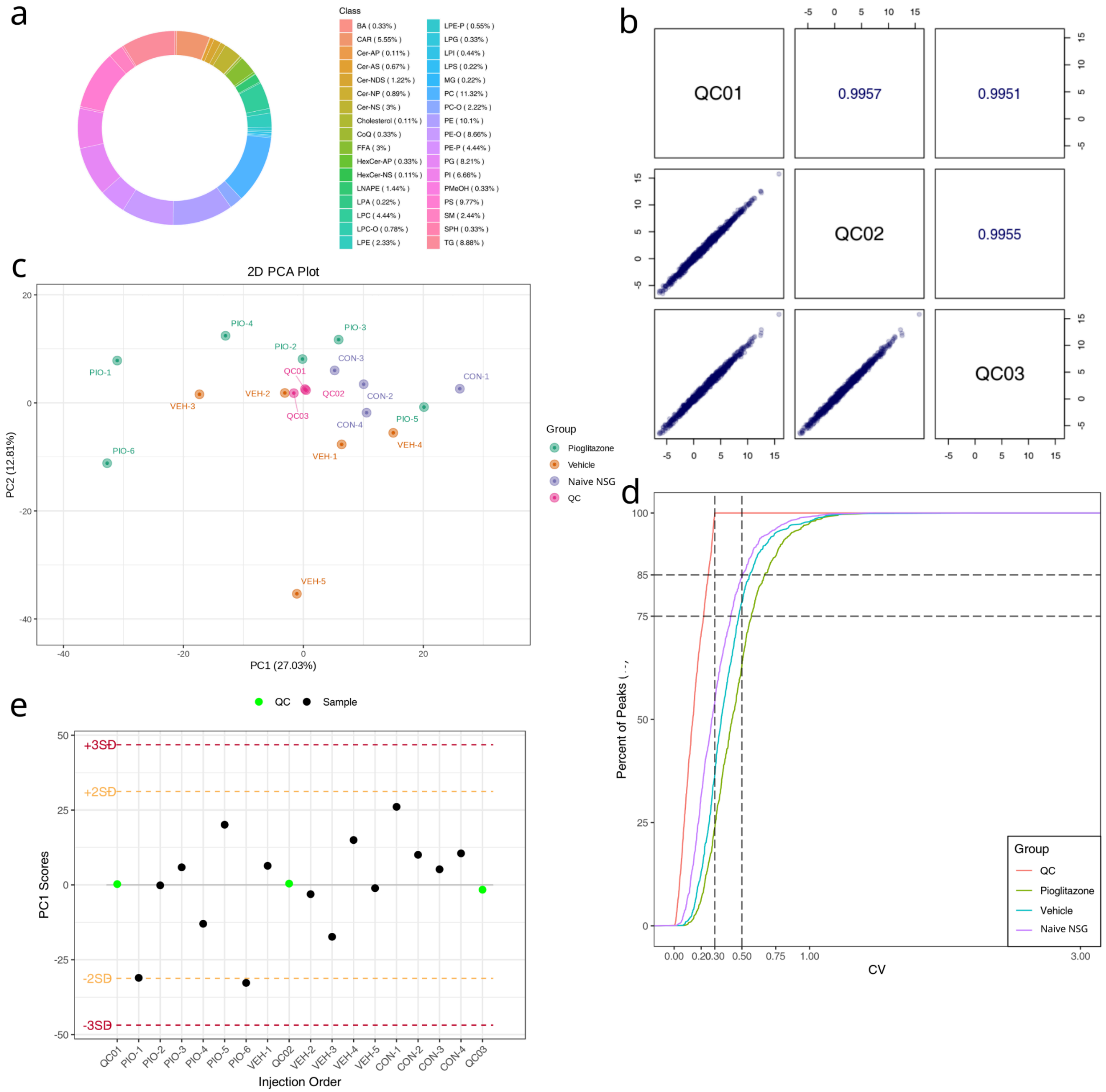

Supplementary Figure S4. Lipidomics OPLS-DA model validations and PCA plots. (**a-c**) Orthogonal partial least squares discriminant analysis (OPLS-DA) model validation for pioglitazone vs. vehicle (**a**), vehicle vs. Naive NSG (**b**), and pioglitazone vs. Naive NSG (**c**). (**d-f**) 2D PCA plot of pioglitazone (green) vs. vehicle (orange) (**d**), vehicle (green) vs. Naive NSG (orange) (**e**), and pioglitazone (green) vs. Naive NSG (orange) (**f**). (**e-f**) Abbreviation CON refers to Naive NSG mice.

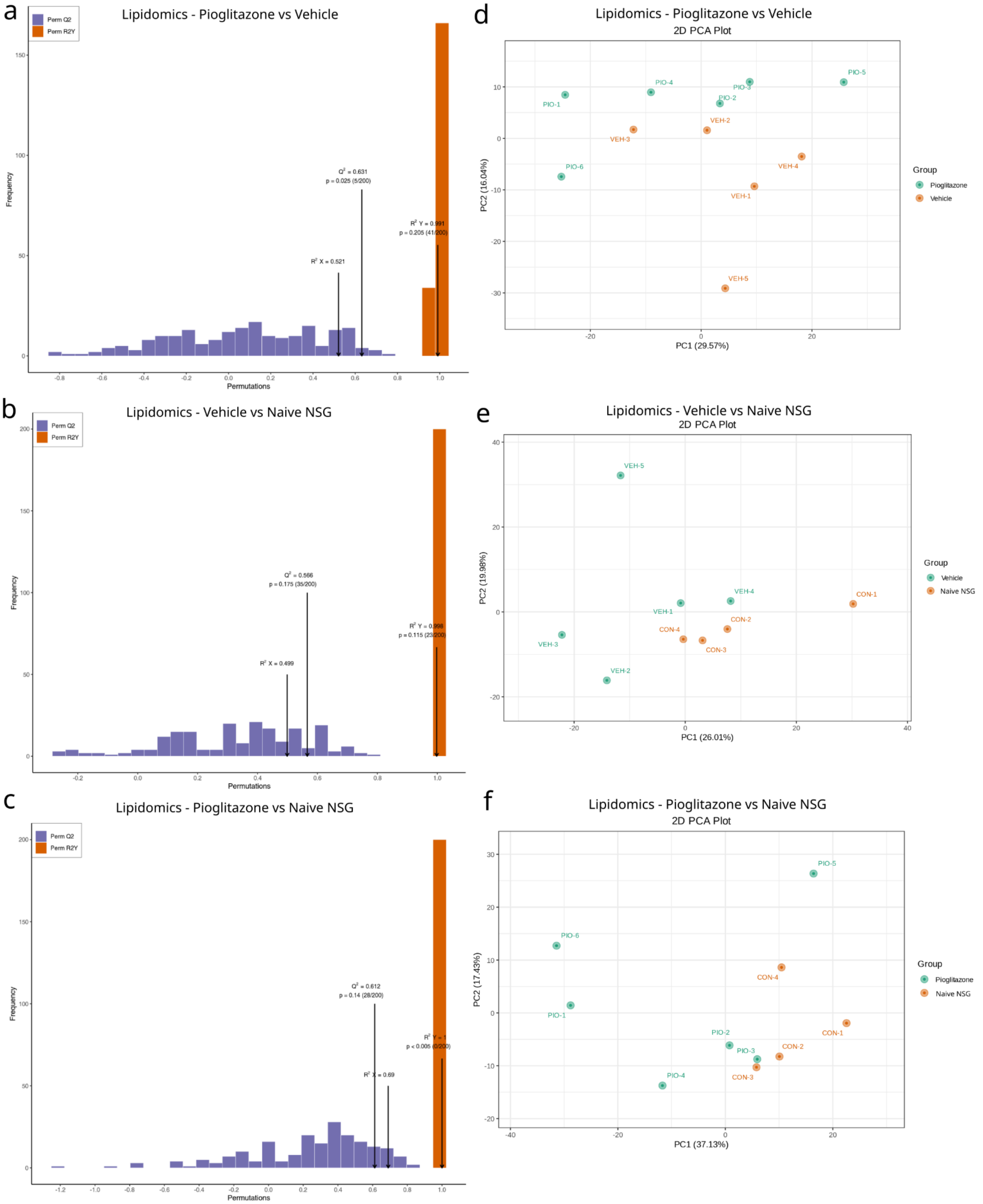

Supplementary Figure S5. Quality control (QC) metrics for untargeted metabolomics. (**a**) Ring plot describing proportion of each compound class combined from all samples. (**b**) Pearson correlation analysis of QC samples. The |r| values in upper right squares represent correlation between QC samples, the closer to 1 the better. (**c**) Two-dimensional principal component analysis (PCA) plot including QC samples. (**d**) Coefficient of variation (CV) plot for QC and sample groups. Horizontal axis represents CV value and vertical axis represents the percent of peaks (proportion of metabolites). (**e**) Principal component 1 (PC1) variation of all samples. Horizontal axis represents injection order of samples and vertical axis represents standard deviation (SD) of PC1 score. (**c,e**) Abbreviation CON refers to Naive NSG mice.

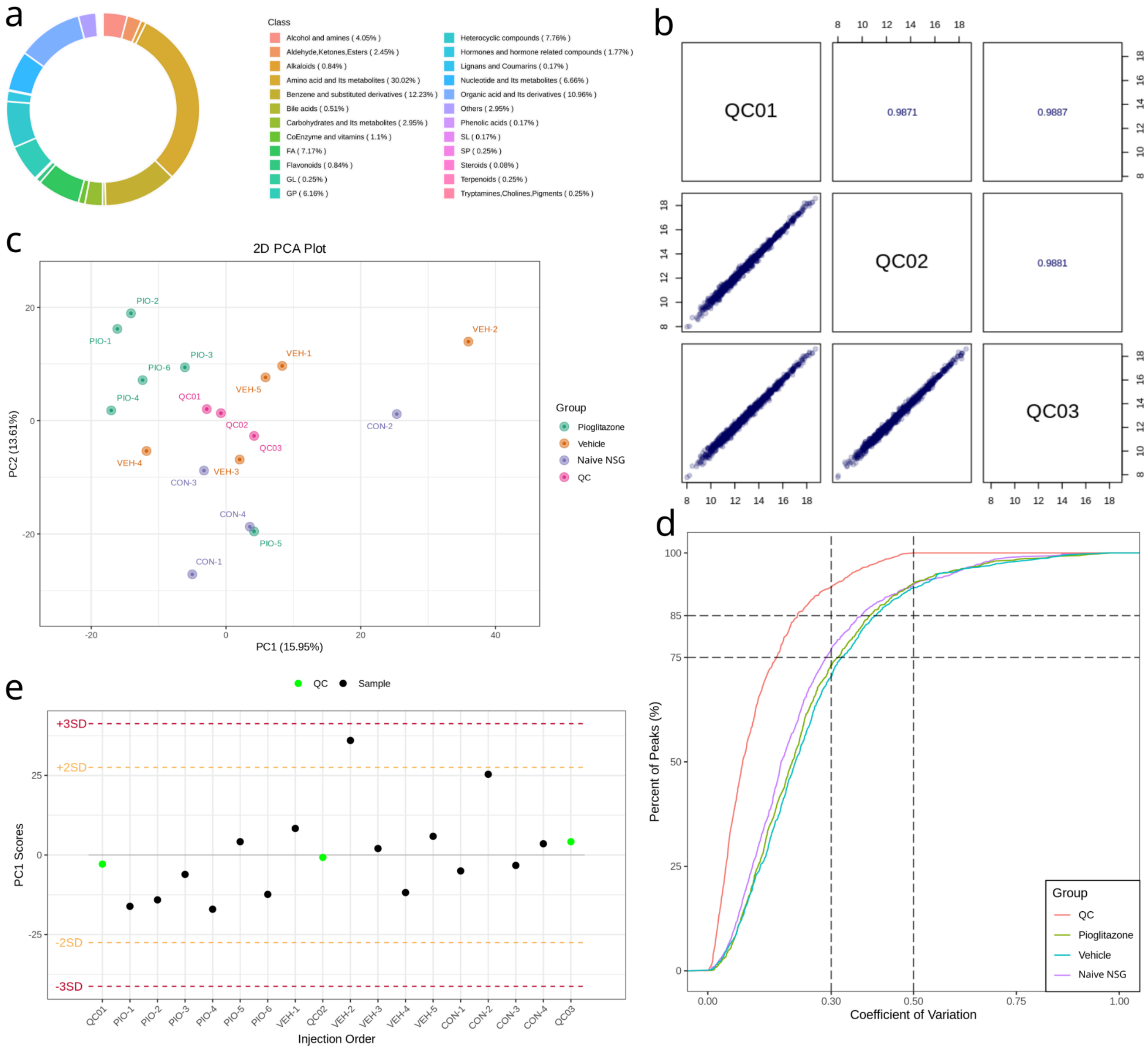

Supplementary Figure S6. Metabolomics OPLS-DA model validations and PCA plots. (**a-c**) Orthogonal partial least squares discriminant analysis (OPLS-DA) model validation for pioglitazone vs. vehicle (**a**), vehicle vs. Naive NSG (**b**), and pioglitazone vs. Naive NSG (**c**). (**d-f**) 2D PCA plot of pioglitazone (green) vs. vehicle (orange) (**d**), vehicle (green) vs. Naive NSG (orange) (**e**), and pioglitazone (green) vs. Naive NSG (orange) (**f**). (**e-f**) Abbreviation CON refers to Naive NSG mice.

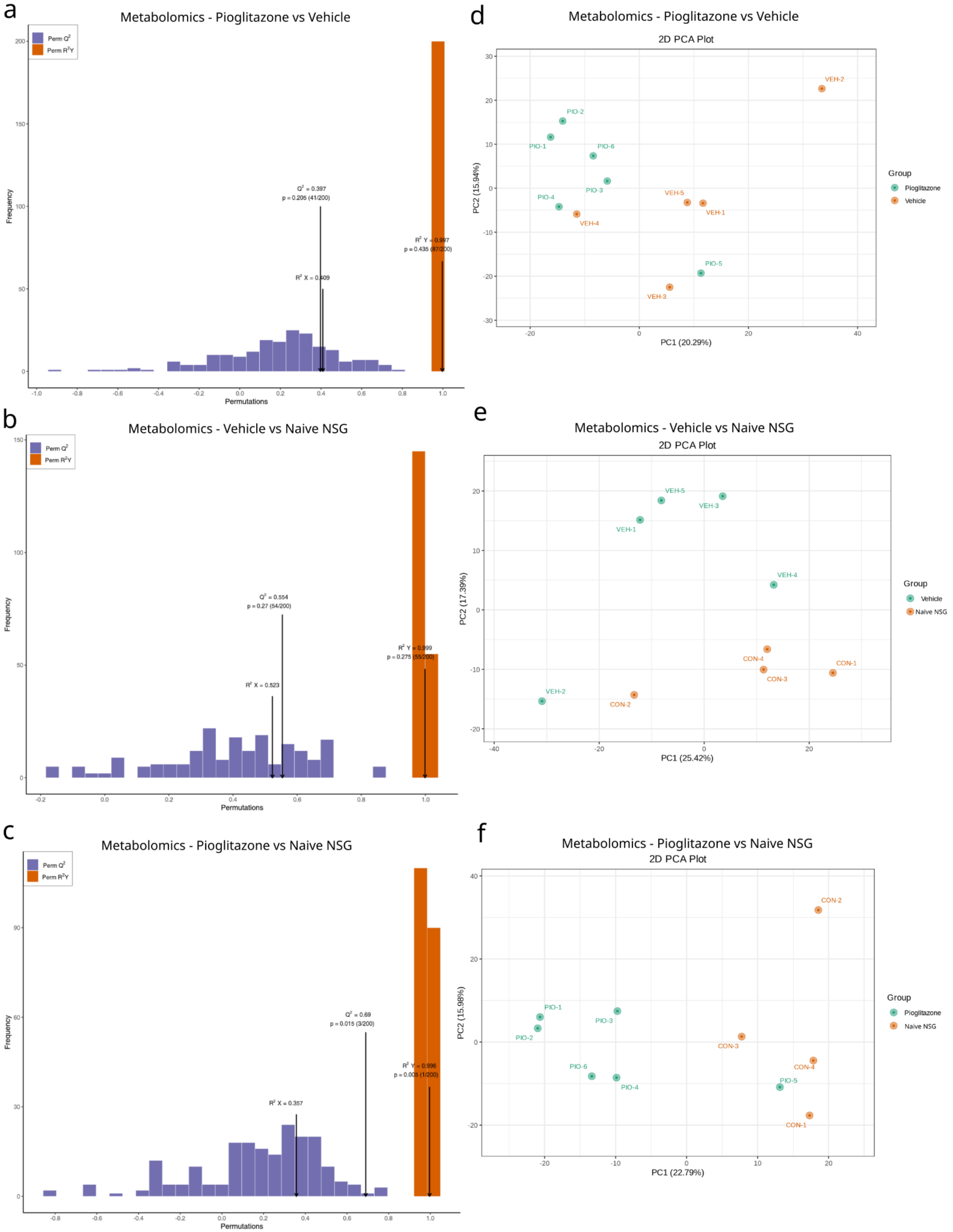

Supplementary Figure S7. Additional pathways from untargeted metabolomics for vehicle vs. Naive NSG (left side) and PioTx vs. vehicle (right side). Asterisks indicate FDR-corrected p values < 0.05. (**a**) Metabolite set enrichment analysis (MSEA) of vehicle vs. Naive NSG. (**b**) MSEA of PioTx vs. vehicle. No FDR-corrected significant *p*-values.

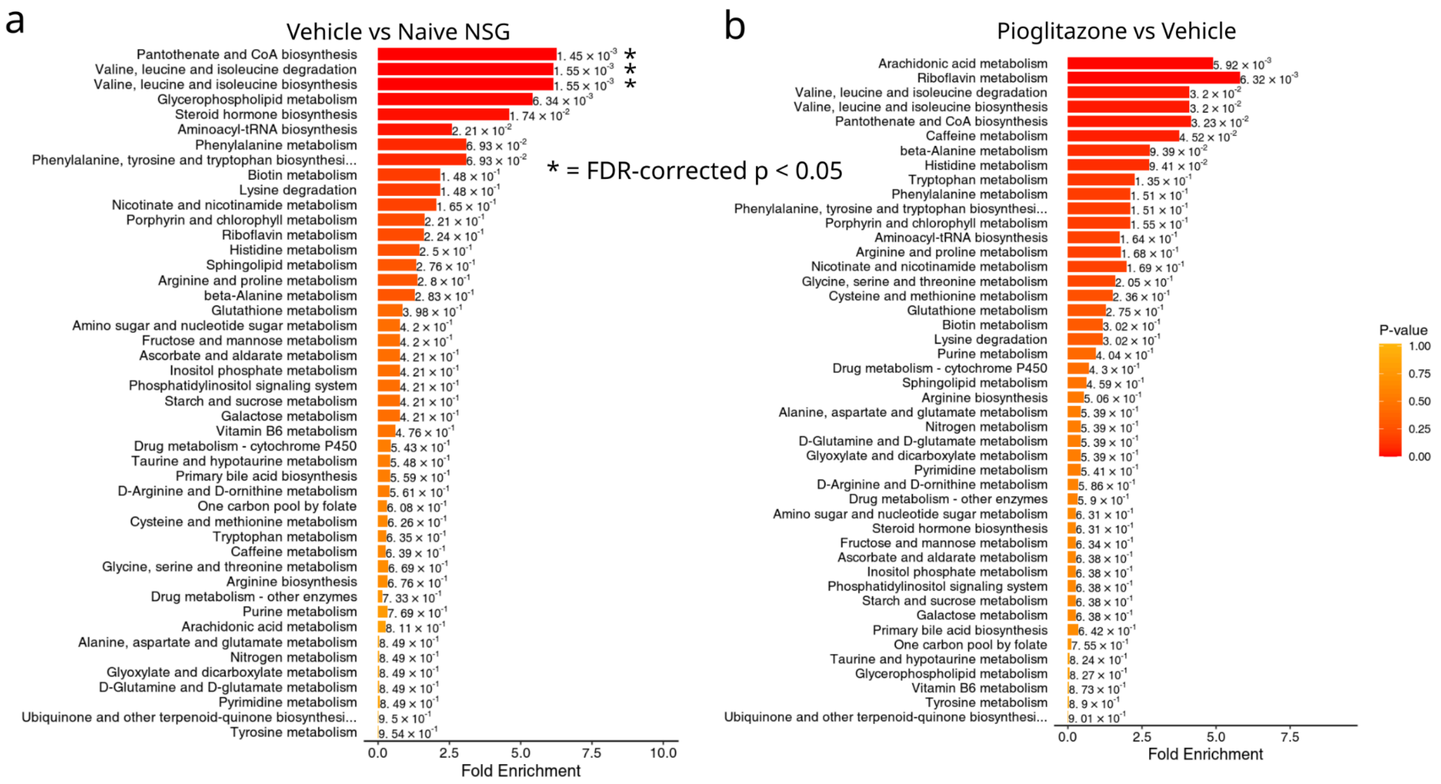

Supplementary Figure S8. *Ex vivo* soleus functional testing. (**a**) Two-way ANOVA analysis of all soleus contractions normalized to first repetition. Plotted as first repetition and every subsequent tenth repetition using normalized data points. (**b**) Area under the fatigue curve (AUC) for data presented in **a**. (**c**) Extensor digitorum longus (EDL) absolute force-frequency relationship (FFR) curve. (**d**) EDL normalized FFR curve. (**e**) Soleus absolute FFR curve. (**f**) Soleus normalized FFR curve. (**a-f**) Black dotted lines represent mean values for a naive NSG mouse. Error bars represent the standard error of the mean (SE). (**d, f**) Muscle force normalized to percent of maximal theoretical force as determined by the P_{max} parameter for each individual animal's FFR regression model.

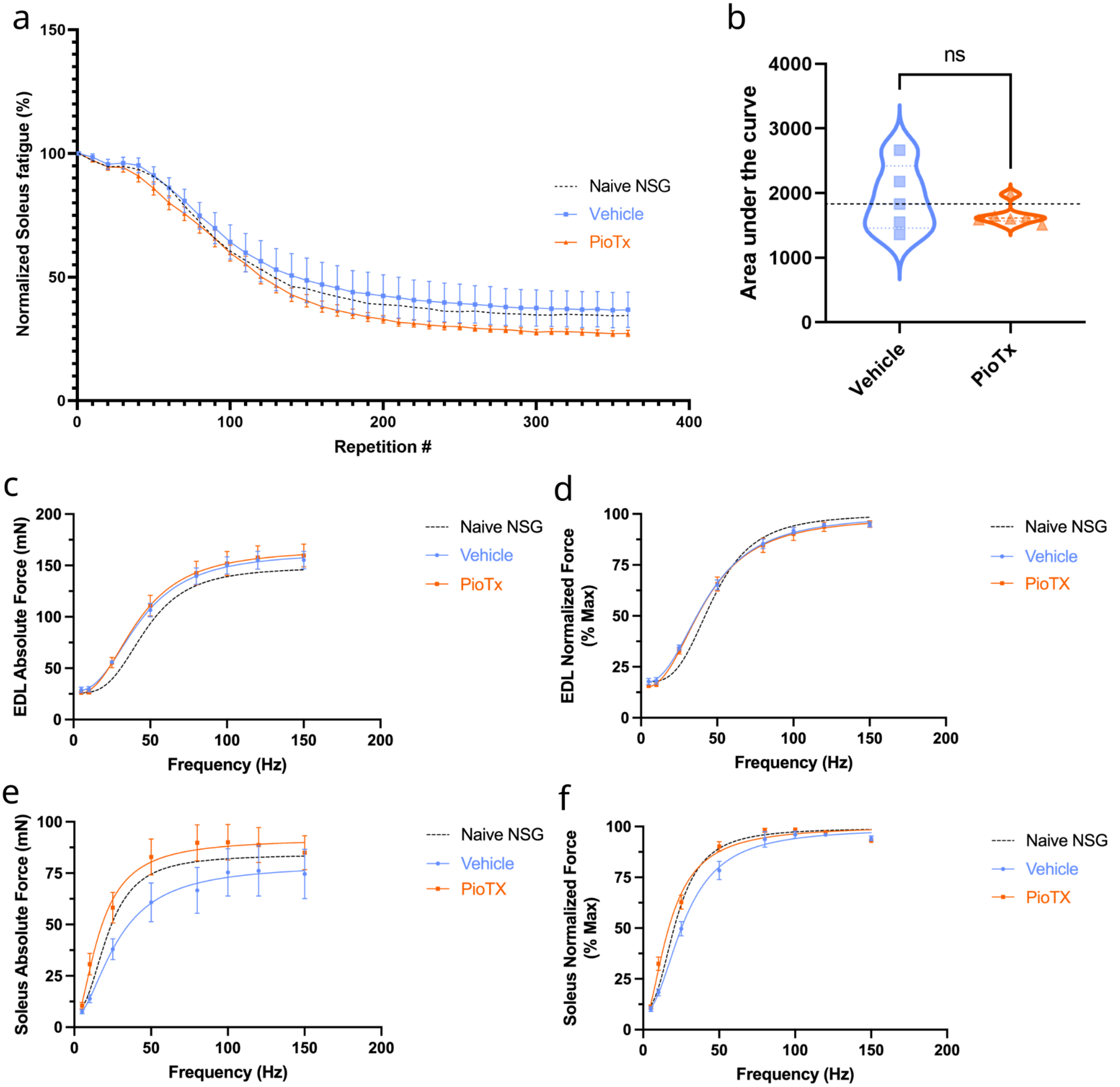

Supplementary Data S1. Untargeted metabolomics internal standard stability in QC samples.

Supplementary Data S2. Cumulative mouse body weights in grams.

Supplementary Data S3. Unnormalized gene expression counts for Pioglitazone and Vehicle groups.

Supplementary Data S4. K-means cluster-5 gene list.

Supplementary Data S5. Pioglitazone- vs. vehicle-treated all adjusted *p*-value significant pathways from KEGG.

Supplementary Data S6. Pioglitazone- vs. vehicle-treated all adjusted *p*-value significant pathways from GO Biological.

Supplementary Data S7. Pioglitazone- vs. vehicle-treated all adjusted *p*-value significant pathways from GO Cellular.

Supplementary Data S8. Pioglitazone- vs. vehicle-treated all adjusted *p*-value significant pathways from GO Molecular.

Supplementary Data S9. Pioglitazone- vs. vehicle-treated all adjusted *p*-value significant pathways from Reactome.
